# Crucial Involvement of IL-6 in Thrombus Resolution in Mice via Macrophage Recruitment and the Induction of Proteolytic Enzymes

**DOI:** 10.3389/fimmu.2019.03150

**Published:** 2020-02-07

**Authors:** Mizuho Nosaka, Yuko Ishida, Akihiko Kimura, Yumi Kuninaka, Akira Taruya, Mitsunori Ozaki, Atushi Tanaka, Naofumi Mukaida, Toshikazu Kondo

**Affiliations:** ^1^Department of Forensic Medicine, Wakayama Medical University, Wakayama, Japan; ^2^Department of Cardiovascular Medicine, Wakayama Medical University, Wakayama, Japan; ^3^Department of Neurological Surgery, Wakayama Medical University, Wakayama, Japan; ^4^Division of Molecular Bioregulation, Cancer Research Institute, Kanazawa University, Kanazawa, Japan

**Keywords:** IL-6, thrombosis, macrophages, matrix metalloproteinases, proteolytic enzymes

## Abstract

After the ligation of the inferior vena cava (IVC) of wild-type (WT) mice, venous thrombi formed and grew progressively until 5 days and resolved thereafter. Concomitantly, intrathrombotic gene expression of *Il6* was enhanced later than 5 days after IVC ligation. IL-6 protein expression was detected mainly in F4/80-positive macrophages in thrombus. When *Il6*-deficient (*Il6*^−/−^) mice were treated in the same manner, thrombus mass was significantly larger than in WT mice. Moreover, the recovery of thrombosed IVC blood flow was markedly delayed in *Il6*^−/−^ compared with WT mice. F4/80-positive macrophages in thrombus expressed proteolytic enzymes such as matrix metalloproteinase (*Mmp*) *2, Mmp9*, and urokinase-type plasminogen activator (*Plau*); and their mRNA expression was significantly reduced in *Il6*^−/−^ mice. Consistently, the administration of anti-IL-6 antibody delayed the thrombus resolution in WT mice, whereas IL-6 administration accelerated thrombus resolution in WT and *Il6*^−/−^ mice. Moreover, IL-6 *in vitro* enhanced *Mmp2, Mmp9*, and *Plau* mRNA expression in WT-derived peritoneal macrophages in a dose-dependent manner; and the enhancement was abrogated by a specific Stat3 inhibitor, Stattic. Thus, IL-6/Stat3 signaling pathway can promote thrombus resolution by enhancing *Mmp2, Mmp9*, and *Plau* expression in macrophages.

## Introduction

Deep vein thrombosis (DVT) is a common vascular disease, often causing post-thrombotic symptoms including pain, heaviness, itching, swelling, and brownish or reddish skin discoloration, with ulcer as its long-term complication (1, 2). Moreover, DVT is frequently associated with pulmonary embolism (PE), one of the major causes of sudden unexpected natural deaths (1, 2). DVT formation has traditionally been thought to be caused by blood stagnancy, endothelial injury of the vein, and hypercoagulability (3). However, evidence is accumulating to indicate the involvement of proinflammatory cytokines and chemokines in the thrombus formation (4–7). Moreover, venous thrombosis resolution and vein wall healing are mediated by leukocytes, particularly macrophages, and their associated chemokines, tissue-type or urokinase-type plasminogen activator (PLAT or PLAU), matrix metalloproteinases (MMPs), and proinflammatory cytokines (8–11). Proinflammatory cytokines can potently activate endothelial cells, can increase the expression of adhesion molecules by endothelial cells, and can promote thrombosis (12, 13). On the contrary, some chemokines can activate leukocytes to accelerate thrombus resolution and intrathrombotic neovascularization (14–16). We previously demonstrated that a proinflammatory cytokine, IFN-γ, can decelerate thrombus resolution by suppressing collagenolysis (17). Moreover, the TNF-α-TNF-Rp55 axis can promote thrombus resolution by inducing macrophages to express *Plau, Mmp2*, and *Mmp9*, the proteolytic enzymes that are crucial to collagenolysis and neovascularization (18). However, the roles of other proinflammatory cytokines in DVT formation and/or resolution remain elusive.

IL-6 is a pleiotropic cytokine involved in inflammation, autoantibody production, vascular permeability, tissue regeneration, metabolism, and hematopoiesis. IL-6 is produced by myriads of cells including T cells, monocytes, fibroblasts, endothelial cells, and keratinocytes (19). IL-6 signaling blockade has recently been proven to be therapeutically effective for a number of autoimmune and inflammatory disorders including rheumatoid arthritis, polymyalgia rheumatica, vasculitis, and Castleman disease, the diseases that exhibit aberrant IL-6 signaling pathway (20). Thrombus forms and resolves with distinct phases consisting of neutrophil and macrophage infiltration, fibrosis, and neovascularization-induced recanalization (14, 21); and the whole process resembles skin wound healing process. We previously revealed that *Il6*^−/−^ mice exhibited delayed skin wound healing with attenuated leukocyte infiltration, re-epithelialization, angiogenesis, and collagen accumulation (22). Thrombus formation and resolution processes share with skin wound healing processes various pathophysiological aspects such as leukocyte accumulation, collagen production, and angiogenesis. This promoted us to conduct our previous study, which revealed markedly enhanced IL-6 protein in thrombi of wild-type (WT) mice (23). However, the pathophysiological roles of IL-6 in venous thrombosis are still unknown.

In this study, we demonstrated that the absence of IL-6 did retard thrombus resolution, together with suppressed expression of MMP-2, MMP-9, and PLAT, compared with that in WT mice. This mirrors the observation that IL-6 could enhance the gene expression of *Plau, Mmp2*, and *Mmp9* in macrophages in an IL-6-dependent manner. These observations implied that the IL-6 can be a target molecule to design the therapeutic strategy for DVT.

## Materials and Methods

### Reagents and Antibodies

Recombinant murine IL-6 (rIL-6) and anti-mouse IL-6 monoclonal antibody (mAb) were purchased from PeproTech (London, UK) and R&D Systems (Minneapolis, MN), respectively. Stattic, a STAT3 inhibitor V, was obtained from Calbiochem (Billerica, MA). The following mAbs and polyclonal Abs (pAbs) were used for immunohistochemical and double-color immunofluorescence analyses: rat anti-mouse F4/80 mAb (Dainippon Pharmaceutical Company, Japan), mouse anti-IL-6 mAb (Santa Cruz Biotechnology, Dallas, Texas), rabbit anti-mouse IL-6 pAbs, rabbit anti-mouse CCL2 pAbs (Novus Biologicals, Centennial, CO), goat anti-mouse MMP-2 pAbs, goat anti-mouse MMP-9 pAbs (Santa Cruz Biotechnology, Dallas, Texas), rabbit anti-mouse PLAU (uPA) pAbs, rabbit anti-mouse tPA pAbs, rabbit anti-mouse PAI-1 pAbs (Santa Cruz Biotechnology, Dallas, TX), mouse anti-mouse Col1A2 mAb (Santa Cruz Biotechnology), rabbit anti-mouse myeloperoxidase (MPO) pAbs (Neomarkers, Fremont, CA), rabbit anti-mouse CD3 mAb (Abcam, Tokyo, Japan), cyanine dye 3-conjugated donkey anti-rat IgG pAbs, cyanine dye 3-conjugated donkey anti-goat IgG, fluorescein isothiocyanate (FITC)-conjugated donkey anti-rat IgG pAbs, and FITC-conjugated donkey anti-rabbit IgG pAbs (Jackson ImmunoResearch Laboratories, West Grove, PA). A Western blot analysis was performed by using the following Abs: rabbit anti-mouse Stat3 mAb, rabbit anti-mouse phosphorylated (p)-Stat3 mAb, rabbit anti-mouse p38 MAPK pAbs, rabbit anti-mouse JNK pAbs, rabbit anti-mouse ERK mAb, rabbit anti-mouse p-p38 MAPK mAb, rabbit anti-mouse p-JNK pAbs, rabbit anti-mouse p-ERK mAb, and rabbit anti-GAPDH mAb (Cell Signaling Technology, Danvers, MA).

### Mice

Pathogen-free male BALB/c mice that are 8–10 weeks old were obtained from Japan SLC (Shizuoka, Japan) and were designated as WT mice in this study. *Il6*^−/−^ mice were a kind gift of Dr. Blüthmann (24) and were back-crossed to BALB/c mice for more than 10 generations. Subsequently, age- and sex-matched *Il6*^−/−^ mice were used in these experiments (25). All mice were housed individually in cages under specific pathogen-free conditions during the experiments. All animal experiments were approved by the Committee on Animal Care and Use in Wakayama Medical University and complied with the Guidelines for the Care and Use of Laboratory Animals of Wakayama Medical University.

### Inferior Vena Cava Ligation-Induced Deep Vein Thrombus Model

Intravenous thrombus formation was induced as previously described (14, 17, 26). In brief, after deep anesthesia with intraperitoneal injection of pentobarbital (50 mg/kg of body weight), a 2-cm incision was made along the abdominal midline. Then, inferior vena cava (IVC) was exposed carefully, and a 21-gauge needle was placed along the exposed IVC. Subsequently, IVC was ligated with the needle using 3-0 silk suture, followed by pulling out the needle. This procedure can induce thrombus formation in almost all the mice. In some experiments, WT mice were intraperitoneally given anti-mouse IL-6 mAb [5 μg/mouse in 200 μl of phosphate-buffered saline (PBS)] at 1, 3, 6, and 8 days after IVC ligation. WT and *Il6*^−/−^ mice were intraperitoneally given rIL-6 (0.3 μg/mouse in 200 μl of PBS) at 1, 4, and 8 days after IVC ligation. At the indicated time intervals after the IVC ligation, mice were euthanized by an overdose of diethyl ether, and intravenous thrombi were harvested for the determination of the weights.

### Histopathological Analyses

At the indicated time intervals after IVC ligation, thrombi were harvested and fixed in 4% formaldehyde buffered with PBS (pH 7.2), and paraffin-embedded sections (4 μm thick) were made. The sections were stained with hematoxylin and eosin (H&E) or Masson trichrome solution.

### Immunohistochemical Analyses

Deparaffinized sections were immersed in 0.3% H_2_O_2_ in methanol for 30 min to eliminate endogenous peroxidase activities. The sections were further incubated with PBS containing 1% normal serum derived from the same species as the origin of the secondary Abs and 1% bovine serum albumin (BSA) to reduce non-specific reactions. The sections were incubated with anti-mouse F4/80 mAb, anti-mouse CCL2 pAbs, anti-mouse Col1A2 mAb, anti-mouse MPO pAbs, anti-mouse CD3 mAb, or anti-mouse IL-6 pAbs at a concentration of 1 μg/ml at 4°C overnight. After the incubation of biotinylated secondary Abs, immune complexes were visualized using Catalyzed Signal Amplification System (Dako) according to the manufacturer's instructions.

### Measurements of Intrathrombotic Leukocytes

Intrathrombotic macrophage and CCL2-positive cell numbers were determined as previously described (17, 18). Briefly, after F4/80-positive macrophages, MPO-positive neutrophils, or CCL2-positive cells were counted in five high power fields (×1,000) within the thrombus, the total numbers in the five fields were combined. All measurements were performed by an examiner without a prior knowledge of the experimental procedures.

### Double-Color Immunofluorescence Analyses

Deparaffinized sections were incubated with PBS containing 1% normal donkey serum and 1% BSA to reduce non-specific reactions as previously described (17). Thereafter, the sections were further incubated with the combinations of anti-F4/80 and anti-IL-6, anti-MMP-2, anti-MMP-9, or anti-PLAU (uPA); anti-MPO and anti-IL-6; or anti-CD3 and anti-IL-6. All Abs were used at a concentration of 1 μg/ml. After the incubation with fluorochrome-conjugated secondary Abs (15 μg/ml) at room temperature for 30 min, the sections were observed under a fluorescence microscopy. In some experiments, nuclei were stained using 4′,6-diamidino-2-phenylindole (DAPI; Roche Diagnostics, Indianapolis, IN) according to the manufacturer's instructions.

### Inferior Vena Cava Blood Flow Measurement by Laser Doppler Flowmeter

At 5, 10, and 14 days after IVC ligation, microvascular IVC blood flow was evaluated by laser Doppler imaging (OMEGAFLO FLO-C1 BV, OMEGAWAVE) as described previously (17). Blood flow through the exposed IVC region of the interest was assessed at three time points; immediately after laparotomy, at the indicated time points after the ligation, and at the harvest. The intensities were reported as the percentage of the baseline blood flow of each animal, in order to ensure consistency.

### Extraction of Total RNAs and Real-Time Reverse Transcription–PCR

Real-time reverse transcription (RT)–PCR was performed as described previously (17). Briefly, total RNAs were extracted from tissue samples (100 μg) using ISOGENE (Nippon Gene, Toyama, Japan) according to the manufacturer's instructions, and 5 μg of total RNAs were reverse-transcribed into cDNA at 42°C for 1 h in 20 μl of reaction mixture containing mouse Moloney leukemia virus reverse transcriptase (PrimeScript, Takara Bio, Kusatsu, Japan) with random 6 primers (Takara Bio). Thereafter, generated cDNA was subjected to a real-time PCR analysis using SYBR® *Premix Ex Taq*™ II kit (Takara Bio) with the sets of specific primers (Table 1). Relative quantity of the target gene expression to *Actb* gene was measured by comparative Ct method.

**Table 1:** Sequences of the primers used for real-time RT-PCR.

**Transcript**	**Sequence**
*Il6*	(F) 5′-CCACTTCACAAGTCGGAGGCTTA-3′
	(R) 5′-GCAAGTGCATCATCGTTGTTCATAC-3′
*Col1*	(F) 5′-ATGCCGCGACCTCAAGATG-3′
	(R) 5′-TGAGGCACAGACGGCTGAGTA-3′
*Mmp2*	(F) 5′-GATAACCTGGATGCCGTCGTG-3′
	(R) 5′-CTTCACGCTCTTGAGACTTTGGTTC-3′
*Mmp9*	(F) 5′-GCCCTGGAACTCACACGACA-3′
	(R) 5′-TTGGAAACTCACACGCCAGAAG-3′
*Plau*	(F) 5′-GAGCAGCTCATCTTGCACGAATAC-3′
	(R) 5′-GCCAGTGATCTCACAGTCTGAACC-3′
*Ccl2*	(F) 5′-GCATCCACGTGTTGGCTCA-3′
	(R) 5′-CTCCAGCCTACTCATTGGGATCA-3′
*Actb*	(F) 5′-CATCCGTAAAGACCTCTATGCCAAC-3′
	(R) 5′-ATGGAGCCACCGATCCACA-3′

*F, forward primer; R, reverse primer; RT, reverse transcription*.

### ELISA for IL-6

At the indicated time intervals, thrombus samples were obtained and homogenized with 0.3 ml of PBS (pH 7.2) containing complete Protease Inhibitor Cocktail (Roche Diagnostics). The homogenates were centrifuged at 12,000 g for 15 min. IL-6 levels in the supernatant were measured using a specific ELISA kit (Murine IL-6 ELISA kit, Diaclone, Besancon Cedex, France), according to the manufacturer's instructions. The detection limit was 10 pg/ml. Total protein in the supernatant was measured with a commercial kit (BCA Protein Assay Kit; Pierce) using BSA as a standard. The data were expressed as IL-6 (ng/ml)/total protein (mg/ml) for each sample.

### Cell Culture

WT mice were i.p. injected with 2 ml of 3% thioglycollate (Sigma-Aldrich), to obtain intraperitoneal macrophages 3 days later as described previously (17). The obtained cells were judged to consist of more than 95% macrophages as determined by flowcytometry (FCM) using anti-F4/80 Ab. The resultant cells were suspended in antibiotic-free Roswell Park Memorial Institute (RPMI) 1640 medium containing 10% fetal bovine serum (FBS) and incubated at 37°C in three 6-well cell culture plates. 2 h later, non-adherent cells were removed, and the medium was replaced. After the cells were incubated for 24 h in the presence of the indicated concentrations of rIL-6 (1,000 U/ml), together with or without anti-mouse IL-6 mAb (5 μg/ml), or Stattic (20 μM), the cells were subjected to subsequent analyses.

### Western Blotting

The obtained macrophages were homogenized with a lysis buffer [20 mM of Tris–HCl (pH 7.6), 150 mM of NaCl, 1% Triton X-100, and 1 mM of EDTA] containing complete Protease Inhibitor Cocktail (Roche Diagnostics) and were centrifuged to obtain lysates. The lysates (equivalent to 30 μg of protein) were electrophoresed in a 10% sodium dodecyl sulfate (SDS)–polyacrylamide gel and were transferred onto a nylon membrane. After the membrane was sequentially reacted with optimally diluted primary Abs and horseradish peroxidase (HRP)-conjugated secondary Abs, the immune complexes were visualized using ECL system (Amersham Biosciences, Pittsburgh, PA). The band intensities were measured using NIH Image Analysis Software version 1.48 (National Institutes of Health).

### Measurement of Prothrombin Time and Activated Partial Thromboplastin Time

Blood samples were taken with 3.8% citrate solution and centrifuged to obtain plasma samples. Prothrombin time (PT) and activated partial thromboplastin time (APTT) of citrated plasma samples were measured by using COAGSEARCH (A&T) according to the manufacturer's instructions.

### Statistical Analyses

Data were expressed as the mean ± SEM. For the comparison between WT and *Il6*^−/−^ mice at multiple time points, a two-way ANOVA followed by Dunnett's *post-hoc* test was used. To compare the values between two groups, unpaired Student's *t*-test was performed. In the series of IL-6 stimulation on peritoneal macrophages *in vitro*, a one-way ANOVA followed by Dunnett's *post-hoc* test was used. *p* < 0.05 was considered statistically significant. All statistical analyses were performed using Statcel3 software under the supervision of a medical statistician.

## Results

### Intrathrombotic IL-6 Expression After the Inferior Vena Cava Ligation

**Figure 1:**
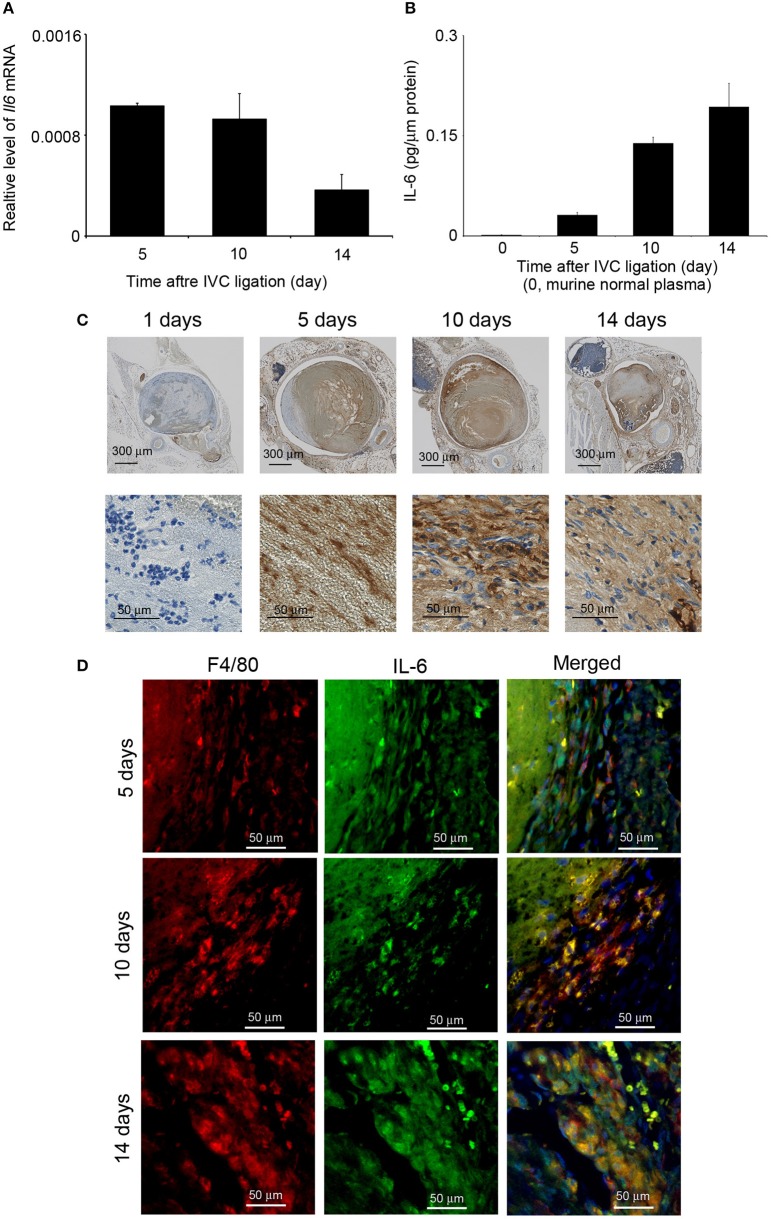
Intrathrombotic expression of IL-6 in wild-type (WT) mice after inferior vena cava (IVC) ligation. **(A)** *Il6* gene expression was examined by real-time reverse transcription (RT)–PCR as described in section Materials and Methods. All values represent mean ± SEM (*n* = 6). **(B)** Intrathrombotic IL-6 protein levels were determined by ELISA. All values represent mean ± SEM (*n* = 6). **(C)** Immunohistochemical analysis of intrathrombotic IL-6 expression (original magnification, ×100, upper panel; ×400, lower panel). **(D)** A double-color immunofluorescence analysis of IL-6-expressing cells in the thrombus. The samples were immunostained with the combination of anti-F4/80 mAb and anti-IL-6 pAbs as described in section Materials and Methods. The fluorescent images were digitally merged in the right panel. Representative results from six independent experiments are shown here [original magnification, ×400; blue, nuclear staining by 4′,6-diamidino-2-phenylindole (DAPI)].

The detection of IL-6 in venous thrombi in autopsy cases (our unpublished data) prompted us to examine intrathrombotic gene expression of *Il6* in WT mice after IVC ligation. *Il6* mRNA was detected in the thrombus 5 days after IVC ligation, and its expression was decreased later than 10 days (Figure 1A). Consistently, IL-6 protein could be detected at day 5 and later (Figure 1B). IL-6 protein was immunohistochemically found in macrophage-like cells inside thrombus (Figure 1C). Consistently, double-color immunofluorescence analyses identified F4/80^+^ macrophages but not MPO^+^ neutrophils and CD3^+^ T cells as a main cellular source of IL-6 (Figure 1D and Figure S1). Thus, these observations would imply the involvement of macrophage-derived IL-6 in the formation and/or resolution of deep vein thrombi.

### Impaired Thrombus Resolution in the Absence of IL-6

**Figure 2:**
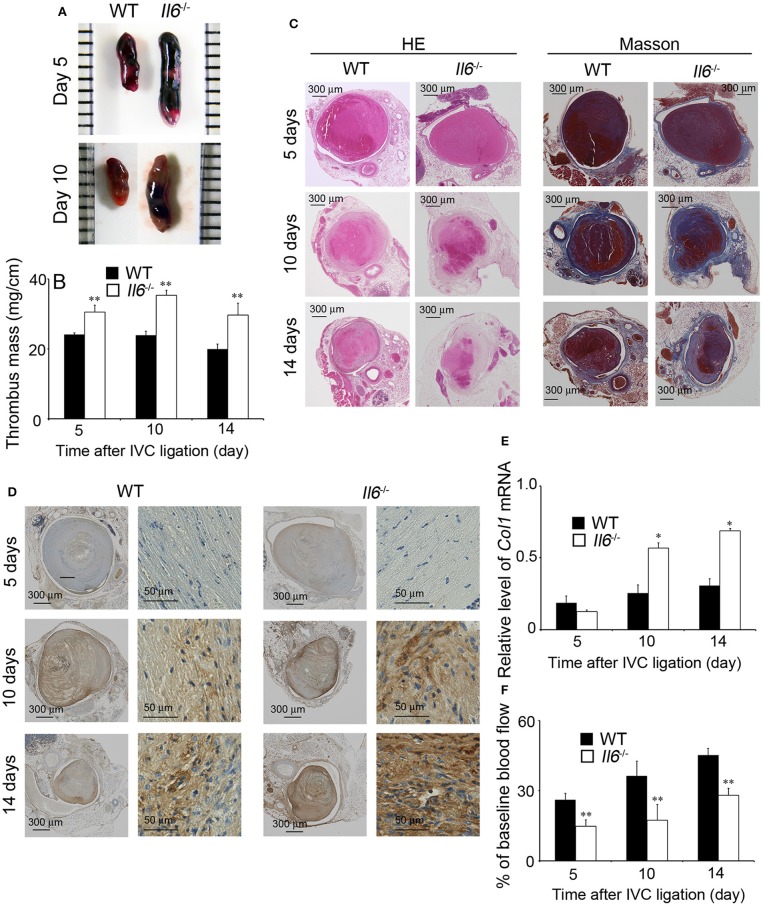
Inferior vena cava (IVC) ligation-induced deep vein thrombus formation in wild-type (WT) and *Il6*^−/−^ mice. **(A)** Macroscopic appearance of venous thrombi in WT and *Il6*^−/−^ mice at 5 and 10 days after IVC ligation. Representative results from six independent animals are shown here. **(B)** Thrombus mass of WT and *Il6*^−/−^ mice at the indicated time intervals after IVC ligation. All values represent the mean ± SEM (*n* = 6). **p < 0.01, WT vs. *Il6*^−/−^. **(C)** Histopathological analyses of venous thrombi obtained from WT and *Il6*^−/−^ mice at 5, 10, and 14 days after IVC ligation. Venous thrombi were stained with hematoxylin and eosin (H&E) or Masson trichrome solution (Masson). Representative results from six independent experiments are shown here (original magnification, ×100). **(D)** Immunohistochemical detection of Col1A2 proteins in the thrombi obtained from WT and *Il6*^−/−^ mice at 5, 10, and 14 days after IVC ligation. **(E)** Intrathrombotic *Col1* gene expression in WT and *Il6*^−/−^ mice at the indicated time intervals after IVC ligation. All values represent the mean ± SEM (*n* = 6). *p < 0.05, WT vs. *Il6*^−/−^. **(F)** Laser Doppler analysis of thrombosed blood flow. All values represent the values mean ± SEM (*n* = 6 animals). **p < 0.01, WT vs. *Il6*^−/−^.

In order to explore the pathophysiological roles of IL-6 in IVC ligation-induced venous thrombus, we compared thrombus formation between WT and *Il6*^−/−^ mice. At 5 days after IVC ligation, *Il6*^−/−^ mice developed a larger thrombus than did WT mice (Figures 2A,B). However, WT and *Il6*^−/−^ mice exhibited similar coagulation functions as revealed by PT (WT, 9.68 ± 0.27 s vs. *Il6*^−/−^, 9.46 ± 0.43 s) and APTT (WT, 36.88 ± 0.66 s vs. *Il6*^−/−^, 34.91 ± 2.38 s). On the contrary, intrathrombotic collagen areas were remarkably enhanced in *Il6*^−/−^ mice compared with WT mice as revealed by both Masson staining and immunostaining for Col1A2 (Figures 2C,D). Consistently, at 10 and 14 days after IVC ligation, intrathrombotic *Col1* mRNA expression was significantly higher in *Il6*^−/−^ mice than WT mice (Figure 2E), indicating that the thrombus in *Il6*^−/−^ mice was replaced with collagen to a larger extent than that in WT mice. Mirroring collagen contents in thrombus, blood flow recovery was delayed in *Il6*^−/−^ mice compared with WT mice (Figure 2F). Collectively, these observations implied that the lack of IL-6 could retard thrombus resolution with excessive collagen deposition.

### Reduced Intrathrombotic Macrophage Infiltration With Attenuated CCL2 Expression

**Figure 3:**
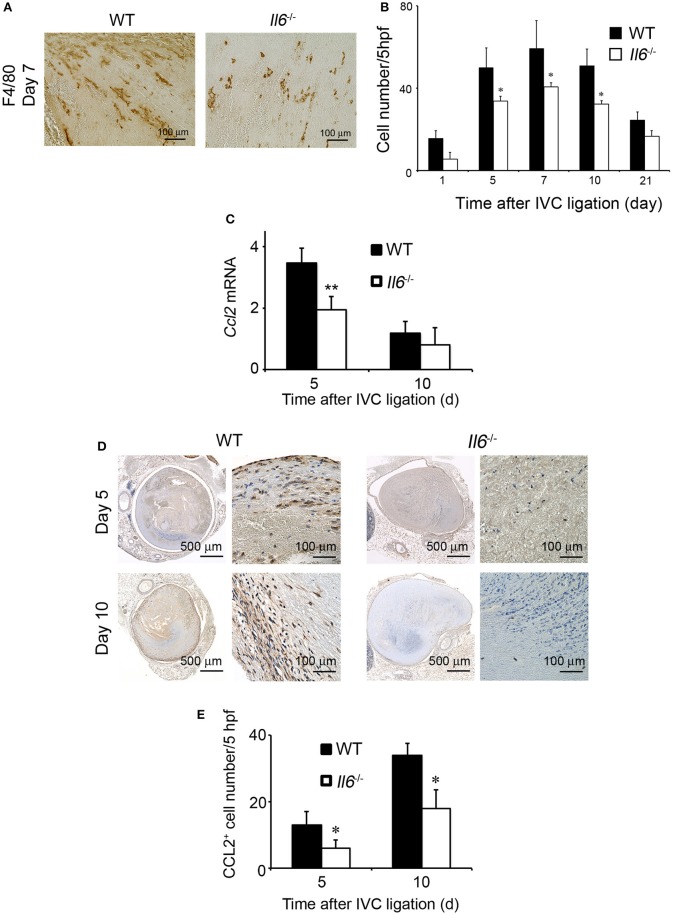
The effects of IL-6 deficiency on macrophage infiltration and CCL2 expression in thrombus tissues. **(A)** Immunohistochemical analysis was performed using anti-F4/80 mAb at day 5 in venous thrombus samples from wild-type (WT) and *Il6*^−/−^ mice (original magnification, ×400). Representative results from six independent experiments are shown here. **(B)** F4/80-positive macrophage numbers were determined as described in section Materials and Methods. All values represent the mean ± SEM (*n* = 6 animals). *p < 0.05, WT vs. *Il6*^−/−^ **(C)** Intrathrombotic expression of *Ccl2* mRNA after inferior vena cava (IVC) ligation was determined by real-time reverse transcription (RT)–PCR as described in section Materials and Methods. All values represent the mean ± SEM (*n* = 6 animals). **p < 0.01, WT vs. *Il6*^−/−^. **(D)** Immunohistochemical analyses of intrathrombotic CCL2. Representative results from six independent experiments are shown here. **(E)** Intrathrombotic CCL2-positive cell numbers were determined. All values represent the mean ± SEM. *p < 0.05, WT vs. *Il6*^−/−^.

Accumulating evidence implicated the crucial roles of infiltrating macrophages in thrombus resolution (27, 28). F4/80-positive macrophages infiltrated into thrombus in WT and *Il6*^−/−^ mice, reaching a maximal level at 7 days and decreasing thereafter, but intrathrombotic macrophage numbers were larger in WT mice than *Il6*^−/−^ mice (Figures 3A,B). CCL2 is a potent chemotactic cytokine for the intrathrombotic recruitment of monocytes/macrophages *via* CCR2 (14). Hence, we examined intrathrombotic CCL2 expression in WT and *Il6*^−/−^ mice. The intrathrombotic expression of *Ccl2* mRNA was up-regulated in WT mice at 5 days after IVC ligation, whereas the enhancement was significantly attenuated in *Il6*^−/−^ mice (Figure 3C). Moreover, immunohistochemical analyses demonstrated that the intrathrombotic CCL2-positive cell numbers were significantly lower at 5 and 10 days after IVC ligation in *Il6*^−/−^ mice than in WT mice (Figures 3D,E). Thus, the lack of IL-6 reduced intrathrombotic macrophage infiltration with attenuated CCL2 expression.

### Reduced Expression of Macrophage-Derived Proteolytic Enzymes in the Absence of IL-6

**Figure 4:**
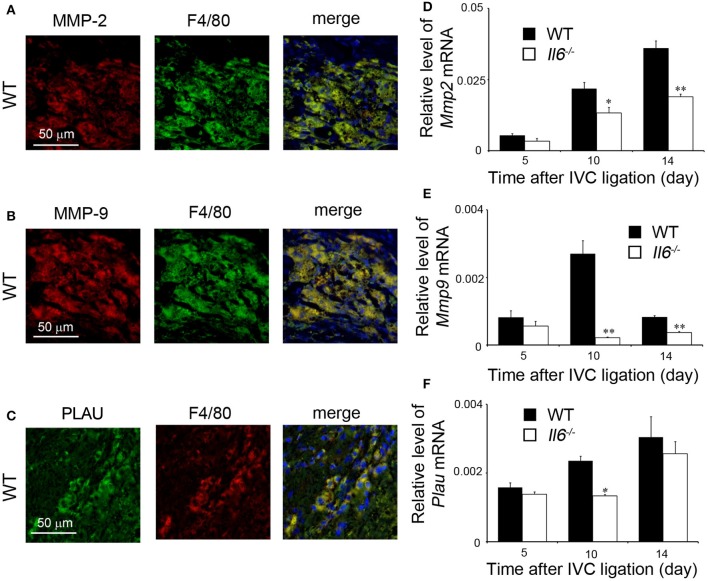
Intrathrombotic expression of proteolytic enzymes in wild-type (WT) and *Il6*^−/−^ mice. **(A–C)** A double-color immunofluorescence analysis of MMP-2-, MMP-9-, or PLAU-expressing cells in the thrombus. The fluorescent images were digitally merged in the right panel. Representative results from six independent experiments are shown here [original magnification, ×400; blue, nuclear staining by 4′,6-diamidino-2-phenylindole (DAPI)]. **(D–F)** Intrathrombotic gene expression of *Mmp2* **(D)**, *Mmp9* **(E)**, and *Plau* **(F)** after inferior vena cava (IVC) ligation. Each gene expression was determined by real-time reverse transcription (RT)–PCR as described in section Materials and Methods. All values represent the mean ± SEM (*n* = 6 animals). *p < 0.05, **p < 0.01, WT vs. *Il6*^−/−^.

We previously revealed that intrathrombotic macrophages were a major source of proteolytic enzymes such as MMP-2, MMP-9, and PLAU, which were essentially involved in thrombus resolution (17, 18). Consistent with our previous reports (17, 18), double-color immunofluorescence analyses revealed that intrathrombotic F4/80-positive macrophages were a main cellular source of MMP-2, MMP-9, and PLAU (Figures 4A–C). Furthermore, later than 10 days after IVC ligation, when thrombus resolution started, mRNA expression of *Mmp2, Mmp9*, and *Plau* was markedly enhanced in thrombus in WT mice, whereas the increments were significantly depressed in *Il6*^−/−^ mice compared with WT mice (Figures 4D–F). These observations would indicate that delayed thrombus resolution in *Il6*^−/−^ mice can be ascribed to depressed expression of *Mmp2, Mmp9*, and *Plau*, the proteolytic enzymes that were expressed mainly by intrathrombotic macrophages.

### Effects of Anti-IL-6 Antibody and Recombinant IL-6 on Thrombus Resolution

**Figure 5:**
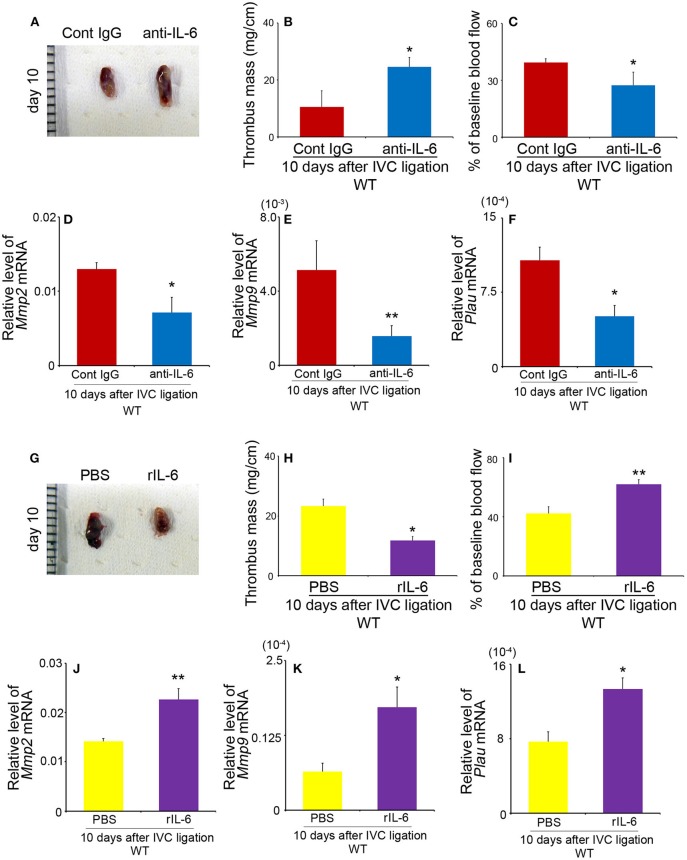
The effects of anti-IL-6 antibody (Ab) and recombinant murine IL-6 (rIL-6) in wild-type (WT) mice on thrombus resolution. **(A–F)** WT mice were intraperitoneally administered with anti-IL-6 as described in section Materials and Methods. **(A)** Macroscopic appearance of venous thrombi obtained from WT mice treated with anti-IL-6 Ab or control IgG at 10 days after inferior vena cava (IVC) ligation. Representative results from six independent animals are shown here. Thrombus weights **(B)** and thrombosed blood flow **(C)** were measured at 10 days after IVC ligation. All values represent the mean ± SEM (*n* = 6 animals). *p < 0.05 vs. control IgG. **(D–F)** Intrathrombotic gene expression of *Mmp2* **(D)**, *Mmp9* **(E)**, and *Plau* **(F)** after IVC ligation. Each gene expression was determined by real-time reverse transcription (RT)–PCR as described in section Materials and Methods. All values represent the mean ± SEM (*n* = 6 animals). *p < 0.05, **p < 0.01, control Ig vs. anti-IL-6. **(G–L)** WT mice were intraperitoneally administered with rIL-6 as described in section Materials and Methods. **(G)** Macroscopic appearance of venous thrombi obtained from WT mice treated with rIL-6 or phosphate-buffered saline (PBS) at 10 days after IVC ligation. Representative results from six independent animals are shown here. Thrombus weights **(H)** and thrombosed blood flow **(I)** were measured at 10 days after IVC ligation. All values represent the mean ± SEM (*n* = 6 animals). *p < 0.05, **p < 0.01, PBS vs. rIL-6. **(J–L)** Intrathrombotic gene expression of *Mmp2* **(J)**, *Mmp9* **(K)**, and *Plau* **(L)** after IVC ligation. Each gene expression was determined by real-time RT-PCR as described in section Materials and Methods. All values represent the mean ± SEM (*n* = 6 animals). *p < 0.05, **p < 0.01, control PBS vs. rIL-6.

We next examined the effects of anti-IL-6 mAb and rIL-6 on the resolution of IVC ligation-induced venous thrombi. Anti-IL-6 mAb significantly increased thrombus weights and decelerated blood flow recovery compared with those in control Ab-treated mice, together with depressed intrathrombotic *Mmp2, Mmp9*, and *Plau* gene expression (Figures 5A–F), similarly as observed in *Il6*^−/−^ mice. On the contrary, when rIL-6 administration started 1 day after IVC ligation, it reduced significantly the thrombus weights and accelerated blood flow recovery with increased intrathrombotic *Mmp2, Mmp9*, and *Plau* gene expression in WT (Figures 5G–L), but without any significant effects on coagulation tests (PT in WT mice: PBS, 9.74 ± 0.28 s vs. rIL-6, 9.85 ± 0.31 s; APTT in WT mice: PBS, 36.6 ± 0.27 s vs. rIL-6, 37.8 ± 3.08 s). These observations would indicate that IL-6 could be therapeutically effective for thrombus formation at least at its early stage. Moreover, supplementation of IL-6 reduced thrombus weights and enhanced blood flow recovery in *Il6*^−/−^ mice (Figure S2). These observations would indicate that IL-6 could regulate the thrombosis resolution without affecting coagulation activities.

### The Effects of IL-6 on Gene Expression of *Mmp2, Mmp9*, and *Plau* in Macrophages

**Figure 6:**
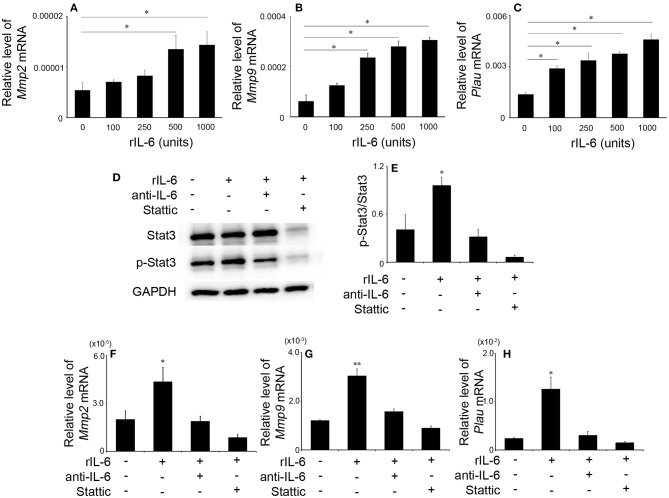
The effects of recombinant murine IL-6 (rIL-6) on the gene expression of *Mmp2, Mmp9*, and *Plau* and on Stat3 signaling in peritoneal macrophages. Peritoneal macrophages were obtained from wild-type (WT) mice and were stimulated as described in section Materials and Methods. The gene expression of *Mmp2* **(A)**, *Mmp9* **(B)**, and *Plau* **(C)** was analyzed by real-time reverse transcription (RT)–PCR. All values represent the mean ± SEM (*n* = 6 independent experiments). *p < 0.05, vs. no stimulation. **(D)** Western blotting analysis using anti-GAPDH pAbs confirmed that an equal amount of protein was loaded onto each lane. Representative results from six independent experiments are shown here. **(E)** The ratios of p-Stat3/Stat3 were densitometrically determined and are shown. All values represent means ± SEM (*n* = 4 independent experiments). **(F–H)** The effects of anti-IL-6 or Stattic on IL-6-induced gene expression of *Mmp2* **(F)**, *Mmp9* **(G)**, and *Plau* **(H)**. Each gene expression was analyzed by real-time RT-PCR. All values represent the mean ± SEM (*n* = 4 independent experiments). *p < 0.05; **p < 0.01, vs. no stimulation.

Depressed intrathrombotic *Mmp2, Mmp9*, and *Plau* expression in *Il6*^−/−^ mice prompted us to examine the effects of IL-6 on the gene expression of *Mmp2, Mmp9*, and *Plau* mRNA expression in WT-derived macrophages. IL-6 significantly enhanced *Mmp2, Mmp9*, and *Plau* mRNA expression in macrophages in a dose-dependent manner (Figures 6A–C). These observations indicated that IL-6-medicated signals regulated *Mmp2, Mmp9*, and *Plau* gene expression in macrophages. Moreover, IL-6 increased significantly phosphorylation of Stat3 (Figures 6D,E), but not that of ERK, p38, and JNK in macrophages (Figure S3). Hence, we further examined the effects of a specific Stat3 inhibitor, Stattic, on IL-6-mediated increases in *Mmp2, Mmp9*, and *Plau* gene expression in macrophages. Stattic abrogated IL-6-induced increases in *Mmp2, Mmp9*, and *Plau* mRNA expression similarly as anti-IL-6 antibody did (Figures 6F–H). Collectively, IL-6 could induce the expression of *Mmp2, Mmp9*, and *Plau* in macrophages through Stat3 pathway.

## Discussion

Accumulating evidence implicates IL-6 as a key regulator of several inflammatory diseases (19). Consistently, we previously revealed that the lack of IL-6 decelerated skin wound healing with a concomitant reduced leukocyte accumulation and collagen deposition (22). Skin wound healing and thrombus resolution share several pathophysiological features: initial platelet aggregation, subsequent leukocyte infiltration, and collagen deposition (26), and final neovascularization. These shared features prompted us to investigate the roles of IL-6 in thrombus formation and resolution. Actually, macrophages were a major source of IL-6 in thrombus, and *Il6*^−/−^ mice exhibited retarded thrombus resolution as evidenced by larger venous thrombi similarly as observed in skin wound healing sites. However, *Il6*^−/−^ mice exhibited increased intrathrombotic collagen contents than did WT mice, suggesting that IL-6 may have distinct roles between skin wound healing and thrombus resolution, in terms of collagen accumulation in the lesions.

Accumulating evidence revealed that various types of leukocytes were crucially involved in thrombus resolution. Neutrophil depletion impaired thrombus resolution (27), whereas intrathrombus injection of peritoneal macrophages accelerated thrombus resolution (28). Moreover, Luther and colleagues demonstrated that the absence of effector memory T cells accelerated thrombus resolution (29). In line with these observations, we previously revealed the potential contribution of intrathrombotic leukocyte accumulation to thrombus resolution (30). Consistently, *Il6*^−/−^ mice exhibited reduced neutrophil (Figure S3) and macrophage accumulation after IVC ligation than did WT mice. Thus, the depressed leukocyte infiltration may contribute to delayed thrombus resolution in *Il6*^−/−^ mice.

Chemokine system, a major controller of leukocyte trafficking, can also regulate thrombus formation and resolution by manipulating the migration of leukocytes, key players in the processes (31). This notion was substantiated by the observations that the administration of exogenous MCP-1/CCL2, a potent macrophage chemoattractant, accelerated venous thrombus resolution together with enhanced F4/80^+^ macrophage infiltration (16). Consistently, the genetic deletion of CCR2 (a specific receptor of CCL2) impaired thrombus resolution with the reduced recruitment of F4/80^+^ macrophages (14). These observations implied that the CCL2–CCR2 axis could promote thrombus resolution by inducing macrophage infiltration into thrombus. Given the observations that IL-6 could up-regulate CCL2 expression in macrophages (32, 33), we determined intrathrombotic CCL2 expression. Macrophages were identified as a major source of CCL2 in thrombus, and intrathrombotic CCL2 expression was significantly attenuated in IVC-ligated *Il6*^−/−^ mice compared with IVC-ligated WT mice. Thus, IL-6 can induce macrophages to express CCL2, which can boost the infiltration of macrophages, the cell component crucially involved in thrombus resolution.

No significant differences were observed in APTT and PTT between WT and *Il6*^−/−^ mice, indicating that coagulation dysfunction can account for the different phenotype of these two strains. On the contrary, *Il6*^−/−^ mice exhibited reduced intrathrombotic gene expression of *Plau*, a major plasminogen activator, whereas IL-6 augmented *Plau* mRNA expression in macrophages, a major cell type present in thrombus. Tissue- and urokinase-type plasminogen activators can promote the generation from plasminogen to plasmin, which has an important role in clotting, fibrinolysis, inflammatory angiogenesis, and tissue remodeling (34). Reflecting the crucial roles of the balance between plasminogen activators and inhibitors in proteolytic and anti-proteolytic activities, the lack of PLAU markedly impaired thrombus resolution (35). Thus, it is probable that IL-6 can promote thrombus resolution by enhancing *Plau* expression in macrophages.

Venous thrombus is replaced by deposited collagen as time passes (26), and therefore, thrombus resolution requires collagen degradation. *Il6*^−/−^ mice displayed enhanced intrathrombotic collagen contents than did WT mice, indicating that IL-6 deficiency can increase collagen synthesis or decrease collagenolysis. The latter possibility was supported by our present observations that *Il6*^−/−^ mice exhibited reduced intrathrombotic expression of MMP-2 and MMP-9, the enzymes that have important roles in collagen turnover during thrombus resolution owing to their potent collagenolysis activities (14, 17). Moreover, evidence is accumulating to indicate that *Mmp2* and *Mmp9* expression can be enhanced by inflammatory cytokines such as IL-1, TNF-α, and IL-6 (33, 36–38). Consistently, we also revealed that IL-6 can augment *Mmp2* and *Mmp9* gene expression in peritoneal macrophages and that these effects were canceled by anti-IL-6 antibody. Thus, IL-6 could be a potent inducer for MMP-2 and MMP-9 in intrathrombotic macrophages.

Several distinct signaling pathways have been presumed to be involved in MMP gene expression (39–41). IL-6 can utilize Stat3 and other MAP kinases to transduce its intracellular signals (19). However, we unraveled that IL-6 significantly enhanced the phosphorylation of Stat3 but not other MAP kinases such as ERK, JNK, and p38, thereby enhancing the mRNA expression of *Mmp2, Mmp9*, and *Plau* in macrophages. Moreover, the Stattic, a Stat3 inhibitor, significantly suppressed IL-6-induced the gene expression of these molecules. Thus, these observations provided the evidence to indicate the crucial involvement of the IL-6/Stat3 signal pathways in venous thrombus resolution through MMP-2, MMP-9, and PLAU.

There are still discrepancies in pathophysiological roles of IL-6 in DVT. Clinically, several inflammatory mediators including IL-6 are proposed to increase the risk of DVT in various types of pathological conditions such as surgery, obesity, cystic fibrosis, sepsis, systemic infection, cancer, inflammatory bowel disease, and lupus (42). Cancer patients, particularly, show a four-fold increased risk for DVT depending on multiple factors such as patient conditions, tumor characteristics, and treatment modalities (43). The presence of the tumor may affect the host coagulation system, and anticancer treatments also increase the risk of venous thromboembolism (VTE) in cancer patients (43). Accumulating evidence implied the close association of IL-6 with the incidence of DVT in cancer patients (44–46). Malaponte and colleagues demonstrated that IL-6 levels in plasma and monocyte samples were higher in cancer patients with DVT than in those without DVT (45). In line with this observation, Stone et al. showed that tumor-derived IL-6 promoted thrombocytosis through the induction of hepatic thrombopoietin, eventually increasing the incidences of DVT in a mouse model of ovarian cancer (44). These observations suggested that IL-6 could promote thrombus formation through an increase of coagulation activity. On the contrary, we observed that IL-6 administration had few effects on coagulation functions.

Thrombosis results from the imbalance caused by increases of formation rate and/or a delay of resolution. However, the previous clinical study on cancer patients did not examine the relationship between IL-6 and resolution-related molecules (44–46). From our observations, IL-6 can promote thrombus resolution by regulating macrophage recruitment *via* the up-regulation of CCL2 and enhancing their expression of MMPs and PLAU in thrombus. In line with this, Malaponte (46) found a positive correlation between IL-6 and MMP-9 plasma concentrations in both DVT and non-DVT cancer patients. These observations may imply that IL-6 expression may be enhanced in the presence of DVT to accelerate thrombus resolution in cancer patients. This assumption has been strengthened by the observations that the administration of exogenous IL-6 accelerated thrombus resolution in WT mice after the IVC ligation.

Anti-coagulant therapy such as warfarin is mainly employed against DVT to prevent pulmonary thromboembolism (PTE), but it increases the incidence of bleeding complications. On the contrary, the administration of IL-6 had few effects on coagulation functions. It is clinically important that IL-6 administration under the presence of thrombus could reduce thrombus size. Our observations implied that IL-6 administration after thrombus formation might be effective for the reduction of thrombus size, because intrathrombotic IL-6 protein levels started to increase at 5 days after the IVC ligation. Thus, IL-6 may be a target molecule to induce DVT resolution, although more work on human clinical conditions is warranted.

## Data Availability Statement

All datasets generated for this study are included in the article/Supplementary Material.

## Ethics Statement

The animal study was reviewed and approved by Wakayama Medical University Animal Care and Use Committees (no. 879).

## Author Contributions

TK and NM formulated the hypothesis and initiated and organized the study. MN and YI performed the main experimental work and analyzed the data. AK, YK, ATar, MO, and ATan helped with some experimental procedures. TK and NM oversaw the experiments, analyzed the data, provided the main funding for the research, and prepared the final manuscript.

### Conflict of Interest

The authors declare that the research was conducted in the absence of any commercial or financial relationships that could be construed as a potential conflict of interest.

## Abbreviations

DVT: deep vein thrombosis
IVC: inferior vena cava
MMP: matrix metalloproteinase
PE: pulmonary embolism
Plau: urokinase-type plasminogen activator.
