# Supplementary material for: Crucial Involvement of IL-6 in Thrombus Resolution in Mice via Macrophage Recruitment and the Induction of Proteolytic Enzymes
Source: Front Immunol. 2020 Feb 7;10:3150. doi: 10.3389/fimmu.2019.03150 (PMC7019028; doi:10.3389/fimmu.2019.03150)
Supplement: Supplementary file 1 [file Data_Sheet_1.pdf]

Supplementary Material

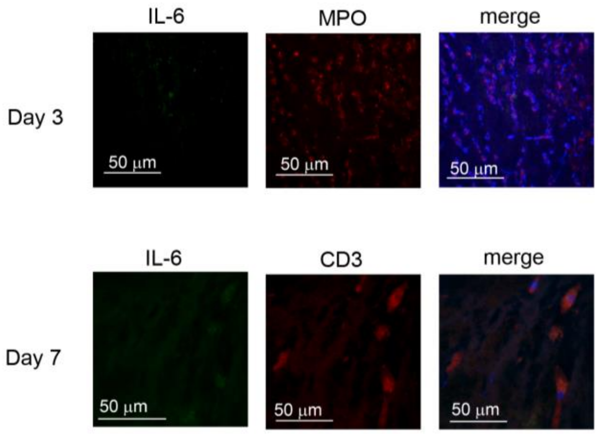

Figure S1. A double-color immunofluorescence analysis of IL-6-expressing cells in the thrombus. The samples were immunostained with the combination of anti-IL-6 and anti-MPO or anti-CD3. The fluorescent images were digitally merged in the right panel. Representative results from six independent experiments are shown here (original magnification, ×400; Blue, nuclear staining by DAPI).

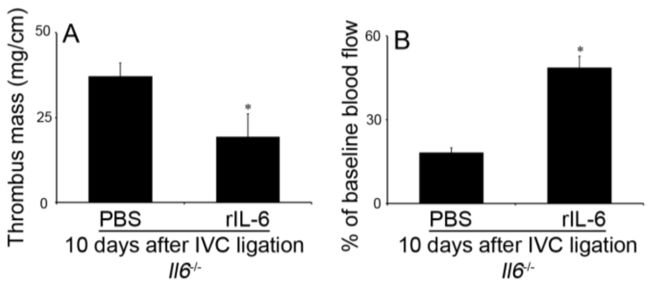

Figure S2. The effects of recombinant IL-6 (rIL-6) in *Il6*$^{-/-}$ mice on thrombus resolution. (A and B) *Il6*$^{-/-}$ mice were intraperitoneally administered with rIL-6 as described in Materials and Methods. Thrombus weights (A) and thrombosed blood flow (B) were measured at 10 days after IVC ligation. All values represent the mean ± SEM (n=6 animals). *, $p < 0.05$, vs. PBS.

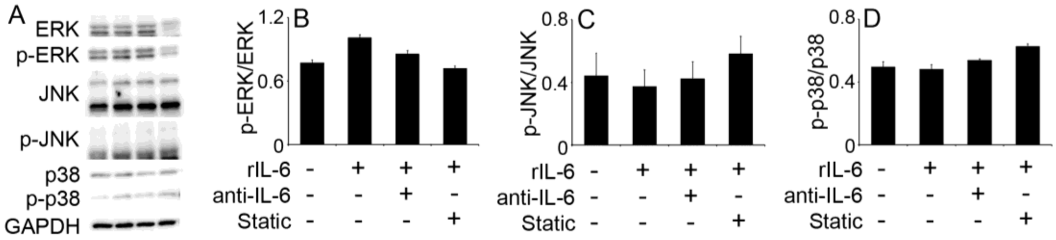

Figure S3. The effects of rIL-6 on MAPK signaling in macrophages. (A) Western blotting analysis using anti-GAPDH pAbs confirmed that an equal amount of protein was loaded onto each lane. Representative results from six independent experiments are shown here. (B-D) The ratios of p-ERK/ERK (B), p-JNK/JNK (C) and p-p38/p38 (D) were densitometrically determined and are shown. All values represent means ± SEM (n=4 independent experiments).

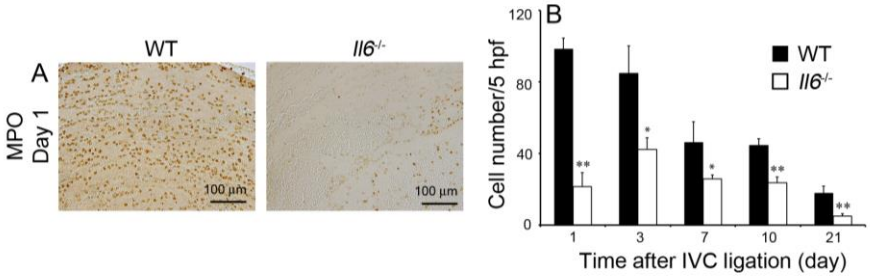

Figure S4. The effects of IL-6 deficiency on neutrophil infiltration in thrombus tissues. (A) Immunohistochemical analysis was performed using anti-myeloperoxidase (MPO) pAb at day 5 in venous thrombus samples from WT and *Il6*$^{-/-}$ mice (original magnification, ×400). Representative results from six independent experiments are shown here. (B) Neutrophil numbers were determined as described in Materials and Methods. All values represent the mean ± SEM (n=6 animals).
